# Unrestricted Ketogenic Diet Feeding Enhances Epithelial Ovarian Cancer Growth In Vivo

**DOI:** 10.3390/nu15122730

**Published:** 2023-06-13

**Authors:** Mariam M. AlHilli, Emily E. Rhoades, Danielle Chau, Surabhi Tewari, Adrian Reich, Alex Myers, Daniel J. Lindner, Justin D. Lathia, Renliang Zhang, Belinda Willard, Gail Cresci, Nathan A. Berger, Ofer Reizes

**Affiliations:** 1 Department of Obstetrics and Gynecology, Division of Gynecologic Oncology, Cleveland Clinic, Cleveland, OH 44195, USA; 2 Department of Cardiovascular and Metabolic Sciences, Lerner Research Institute, Cleveland Clinic, Cleveland, OH 44196, USA; 3 Case Comprehensive Cancer Center, Cleveland, OH 44106, USA; 4 Cleveland Clinic Lerner College of Medicine, Cleveland Clinic, Cleveland, OH 44195, USA; 5 Department of Bioinformatics, Florida Research and Innovations Center, Cleveland Clinic, Port St. Lucie, FL 34987, USA; 6 Department of Cancer Biology, Lerner Research Institute, Cleveland Clinic, Cleveland, OH 44196, USA; 7 Department of Translational Hematology Oncology Research, Cleveland Clinic, Cleveland, OH 44195, USA; 8 Proteomics and Metabolic Core, Lerner Research Institute, Cleveland Clinic, Cleveland, OH 44196, USA; 9 Department of Gastroenterology, Hepatology and Nutrition, Digestive Disease and Surgery Institute, Cleveland Clinic, Cleveland, OH 44195, USA; 10 Department of Inflammation and Immunity, Lerner Research Institute, Cleveland Clinic, Cleveland, OH 44196, USA; 11 Department of Medicine, Division of Hematology and Oncology, Case Western Reserve University School of Medicine, Cleveland, OH 44106, USA

**Keywords:** ketone bodies, high fat diet, ovarian cancer, mice, tumor metabolism, tumor progression, RNA sequencing, dietary intervention

## Abstract

The ketogenic diet (KD) is hypothesized to impact tumor progression by altering tumor metabolism. In this study, we assessed the impact of an unrestricted KD on epithelial ovarian cancer (EOC) tumor growth, gene expression, and metabolite concentration in a mouse model. ID8 EOC cells, which were syngeneic with C57Bl/6J mouse strain and transfected with luciferase (ID8-luc), were injectedand monitored for tumor development. Female mice were fed either a strict KD, a high fat/low carbohydrate (HF/LC) diet, or a low fat/high carbohydrate (LF/HC) diet (n = 10 mice per group) ad libitum. EOC tumor growth was monitored weekly, and tumor burden was determined based on luciferase fluorescence (photons/second). At the endpoint (42 days), tumors were collected and processed for RNA sequencing. Plasma and tumor metabolites were evaluated using LC-MS. The KD-fed mice exhibited a statistically significant increase in tumor progression in comparison to the HF/LC- and LF/HC-fed groups (9.1 vs. 2.0 vs. 3.1-fold, respectively, *p* < 0.001). The EOC tumors of the KD-fed mice exhibited significant enrichment of the peroxisome proliferator-activated receptor (PPAR) signaling and fatty acid metabolism pathways based on the RNA sequencing analysis when compared to the LF/HC- and HF/LC-fed mice. Thus, unrestricted KD diet enhanced tumor progression in our mouse EOC model. KD was associated with the upregulation of fatty acid metabolism and regulation pathways, as well as enrichment of fatty acid and glutamine metabolites.

## 1. Introduction

Epithelial ovarian cancer (EOC) is the most lethal gynecologic malignancy. More than 75% of patients with an advanced disease will experience recurrence after their initial treatment, and most patients will require lifelong therapy to manage their disease. While the genomic landscape of EOC has become better understood in the last decade, knowledge about metabolic and dietary factors that promote or prevent EOC is under-investigated [1]. Patients often resort to self-altering their dietary habits upon the diagnosis of cancer as it is commonly believed that certain dietary interventions may curtail cancer progression [2]. Much of this thinking stems from the fact that cancer cells undergo metabolic reprograming and adapt to oxidative stress, thereby allowing glucose to fuel continuous tumor growth [3]. This hallmark of cancer, also known as the Warburg effect, has emerged as a therapeutic target with accumulating evidence supporting the inhibition of glycolytic metabolism as a tumor-suppressive mechanism [4,5].

Dietary interventions that inhibit tumor glycolytic metabolism, such as ketogenic diet (KD), have gained popularity among cancer patients due to accumulating data showing anti-inflammatory, anti-proliferative, and anti-angiogenic benefits [6,7]. KD has been shown to improve survival, reduce tumor growth, and work in combination with cancer therapies in a variety of cancers, including malignant glioma, colon cancer, gastric cancer, and prostate cancer [6,8,9]. A strict KD consisting of 90% fat, 2% carbohydrates, and 8% proteins induces a metabolic state of glucose depletion, ketone body elevation (ketogenesis), increased fatty acid oxidation, and altered amino acid metabolism [10,11,12]. Some of the proposed anti-tumor benefits of KD include inhibition of the insulin/insulin growth factor (IGF) pathway, alteration in cancer stem cell properties, and upregulation of metabolic signaling pathways, such as AMP kinase activation [6,13].

There is controversy around the tumor-specific propensity of various cancers to respond to KD. A recent study in melanoma showed that KD promoted anti-tumor responses regardless of genetic background, metabolic signature, or immune status [14]. Similarly, in brain tumors, where cells are dependent on glucose for metabolism and display a strong tendency toward the Warburg effect, studies have consistently shown anti-tumor benefits of KD [5,15,16]. Other studies have highlighted the tumor-growth promoting effects of KD, and the poor outcomes in some cancer models, including breast cancer and renal cell carcinoma, have brought to light the issue that KD effects may vary by cancer type [17,18]. Zhang et al. showed that the utilization of ketone bodies for tumor growth requires the presence of four ketolytic enzymes, namely D-beta hydroxybutyrate dehydrogenases 1 and 2 (BDH1 and BDH2) and 3-oxoacid CoA transferases 1 and 2 (OXCT1 and OXCT2) [19]. A low expression of these enzymes is associated with reduced tumor proliferation and sensitization to KD as seen in PANC-1 cells (low expression of ketolytic enzymes) compared to HeLa cells (high expression of ketolytic enzymes) [19]. In experimental models of glioblastoma, which have traditionally shown response to KD, the administration of an unrestricted KD did not decrease tumor growth or improve survival [20]. However, the inhibition of a rate-limiting enzyme of fatty acid oxidation, carnitine palmitoyltransferase (CPT1), altered the growth of glioblastoma cells in vitro, thus highlighting the influence of enzyme expression on the utilization of fatty acids for fuel [20]. These data indicate the need to uncover the biological effects of KD and the specific diet-induced endogenous effects resulting from ketogenesis on tumor growth prior to advocating for specific dietary interventions in cancer therapy. 

Clinical studies on KD in EOC, including a randomized clinical trial that compared patients on a KD to the standard American Cancer Society diet, showed that patients on a KD had improved insulin sensitivity, more favorable body composition, and decreased food cravings. Improvements in fatigue, physical function, and quality of life were also reported [21,22]. Although these studies provide evidence for the positive clinical benefits of KD in patients with EOC, the therapeutic effects of KD have not yet been investigated in EOC. A recent review indicates that KD remains questionable with little evidence to support its efficacy in the treatment of cancers [23]. To study the effects of overt dietary manipulation, we tested three dietary fat modifications in a preclinical EOC mouse model to examine their effects on tumor growth and response to chemotherapy, as well as tumor transcriptomics and metabolomics. 

## 2. Materials and Methods

### 2.1. Cell Lines

ID8 is a cell line derived from mouse ovarian surface epithelial cells that have the ability to form extensive peritoneal tumors in vivo and have no known genetic mutations [24]. ID8 cells have variable ability to perform oxidative phosphorylation and glycolysis (glutamine independent) [25]. The ID8 cells used in this study were a gift from Dr. Vince Tuohy. 

ID8 (syngeneic) EOC cell lines were cultured in Dulbecco’s Modified Eagle Medium (DMEM) containing 5% heat-inactivated FBS (Atlas Biologicals Cat # F-0500-D, Lot F31E18D1), which were grown under standard conditions. For luciferase transduction, in short, HEK293T/17 (ATCC CRL-11268) cells were plated and co-transfected with Lipofectamine 3000 (L3000015 Invitrogen, Waltham, MA, USA), third-generation packaging vectors pRSV-REV #12253, pMDG.2 #12259, and pMDLg/pRRE #12251 (Addgene, Watertown, MA, USA), and a lentiviral vector directing the expression of luciferase reporter pHIV, Luciferase #21375, at 4.5 µg (Addgene, Watertown, MA, USA). Viral particles were harvested, filtered through a 0.45 µm Durapore PVDF Membrane (Millipore Sigma, St. Louis, MO, USA), and added to each cell line’s culture medium. Viral infections were carried out over 72 h and the transduced cells were selected based on their resistance to 2 μg/mL of puromycin (MP Biomedicals, Santa Ana, CA, USA) [26]. 

### 2.2. Animal Studies

**Scheme 1:**
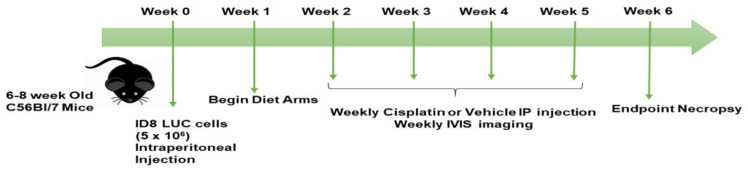
Study scheme and timeline of treatment by week.

Female C57BL/6J (BL/6) mice were purchased from Jackson Laboratories (Bar Harbor, ME) at 6–8 weeks of age. The experimental animals were housed in groups and handled in accordance with the Cleveland Clinic Lerner Research Institute’s Institutional Animal Care and Use Committee (IACUC) approved protocol #2018-2003. The mice were injected intraperitoneally (IP) with 5 × 10^6^ ID8 luciferase-tagged cells (ID8 LUC) after a two-week acclimatization period. The mice (n = 10 per group) were then randomized into different diet arms (high fat/low carbohydrate diet (HF/LC), low fat/high carbohydrate (LF/HC) diet, or KD) at 1 week post cell injection, at the point of measurable disease using an in vivo imaging system (IVIS). The mice were fed ad libitum (Scheme 1). All mouse diets were irradiated and provided by Tekland Envigo. Table 1 shows the diet compositions of the three experimental diet arms. Weekly imaging was performed using the in vivo imaging system (IVIS) and cisplatin or vehicle/placebo intraperitoneal (IP) injection was performed at weeks 1–5, followed by endpoint necropsy at week 6 post ID8 LUC cell injection. Weekly blood glucose and ketone levels were also assessed following tail vein blood draw using a standard laboratory glucometer (Precision Xtra^®^ (Abbott, Abbott Park, IL, USA)). Mouse weight and body composition were assessed weekly using EchoMRI and a standard laboratory scale. The cisplatin-treated groups received 5 mg/kg of cisplatin weekly at the point of imaging using the IVIS via IP injection. The vehicle-treated mice were injected with 1× PBS. 

### 2.3. Mouse Body Composition Scoring (BCS)

The body composition (fat mass and lean mass) of unanesthetized mice was assessed using EchoMRI body composition analysis (EchoMRI, Houston, TX, USA). The mice’s wellbeing was assessed directly: while gently restraining a mouse by holding the base of its tail, the observer (blinded to the treatment group) used the thumb and index finger of the other hand to palpate the degree of muscle and fat over the sacroiliac region. A score from 1 to 5 was given to each mouse weekly following IP tumor cell injection based on previous literature [27]. The same observer was used for all tests. Based on the established IACUC protocols #2018-2003, a BCS of 2 or lower was defined as meeting the endpoint criteria for euthanization.

### 2.4. Tumor Monitoring via Imaging Using 2D IVIS

All bioluminescence images were taken with IVIS Lumina (PerkinElmer, Waltham, MA) using D-luciferin as previously described [26]. The mice received an IP injection of D-luciferin (Goldbio LUCK-1G, 150 mg/kg in 150 µL) under inhaled isoflurane anesthesia. The images were analyzed (Living Image Software 4.8.0), and total flux was reported in photons/second/cm^2^/steradian for each mouse abdomen. All images were obtained with automatic exposure. 

### 2.5. Tumor RNA Sequencing

ID8 tumors were collected from the mice during euthanasia after treatment with vehicle HF/LC, LF/HC, and KD and were snap-frozen in liquid nitrogen. Prior to RNA extraction (Takara Nucleospin, Takara Bio, San Jose, CA, USA), the tumors were crushed using ice-cold mortar and pestle under sterile conditions. Following RNA isolation, Nanodrop was used to measure the absorption at 260/280 nm to assess the quality and quantity of the collected RNA. The cDNA libraries were prepared by the LRI Genomics Core facility and sent to Macrogen Inc. (Seoul, South Korea) for RNA sequencing. The RNA sequencing results were analyzed for 3 samples per treatment group by the Department of Bioinformatics at the Florida Research and Innovation Center, Cleveland Clinic, Florida. The reads were trimmed of exogenous adapter sequences and low-quality bases using cutadapt (version 2.8) [28]. The trimmed reads were then mapped to the mouse genome (ENSEMBL GRCm38, release 101) and the features were counted using STAR (version 2.7.5a) [29]. DESeq2 (version 1.30.1) [30], R (version 4.0.4), and custom scripts were used to test for differential gene expression. Gene set enrichment analysis (GSEA) was performed using the GSEA analyzer (version 4.1.0) [31,32]. All sequencing data were deposited into the Gene Expression Omnibus NCBI database (BioProject ID PRJNA976354)

### 2.6. Metabolomics

For targeted fatty acid (FA) metabolomics analysis, metabolites were extracted from eleven mouse plasma and tissue samples (7 KD and 4 HF/LC) using methanol/water/chloroform spiked with the internal standard FA (21:5). The samples were dried and reconstituted in 0.2% acetic acid in Methanol/Acetonitrile (1:1) for analysis using liquid chromatography–mass spectrometry (LC-MS) at the Lerner Research Institute metabolomics core. A 5 µL aliquot of these samples were injected into a Gemini C18 (3 µm, 2 × 150 mm, Phenomenex (Torrence, CA, USA) column for reverse-phase chromatographic separation. Fatty acid metabolites were eluted using a gradient of mobile phase A (water containing 0.2% acetic acid) and mobile phase B (containing 0.2% acetic acid in Methanol/Acetonitrile (1:1)) at a flow rate of 0.3 mL/min. The gradient started at 75% of mobile phase B for 2 min, followed by a linear increase to 100% of mobile phase B in 6 min. The high-throughput liquid chromatography (HPLC) eluent was directly injected into a Thermo Scientific Quantiva triple quadrupole mass spectrometer, and fatty acids were ionized using negative-ion electrospray ionization (ESI). The collected fatty acid metabolites were analyzed using the Multiple Reaction Monitoring (MRM) mode with the following transitions; FA (14:0): 227>227, FA (16:0): 255>255, FA (16:1): 253>253, FA (16:2): 251>251, FA (18:0): 283>283, FA (18:1): 281>281, FA (18:2): 279>279, FA (18:3): 277>277, FA (18:4): 275>275, FA (20:0): 311>311, FA (20:1): 309>309, FA (20:2): 307>307, FA (20:3): 305>305, FA (20:4): 303>303, FA (20:5): 301>301, FA (20:6): 299>299, FA (21:5): 315>315 (internal standard), FA (22:0): 339>339, FA (22:1): 337>337, FA (22:2): 335>335, FA (22:3): 333>333, FA (22:4): 331>331, FA (22:5): 329>329, FA (22:6): 327>327, FA (24:0): 367>367, FA (24:1): 365>365, FA (24:2): 363>363, FA (24:3): 361>361, and FA (24:4): 359>359. The peak areas for the fatty acid compounds and the internal standard were integrated using the software TraceFinder 3.1 (Thermo Scientific, Waltham, MA, USA). The internal standard calibration curves were used for the quantitation of fatty acid compounds, and the levels were normalized to the protein content.

A similar method was used for amino acid metabolite extraction. The samples were dried and reconstituted in 0.2% acetic acid in water. A 2 µL aliquot of these samples were injected into a Gemini C18 (3 µm, 2 × 150 mm, Phenomenex) column for reverse-phase chromatographic separation. The amino acids were eluted using a gradient of mobile phase A (water containing 0.2% acetic acid + 10 mM of Ammonium Acetate) and mobile phase B (Methanol with 0.2% acetic acid + 10 mM of Ammonium Acetate) at a flow rate of 0.3 mL/min. The gradient started at 0% of mobile phase B for 2 min, followed by a linear increase to 100% of mobile phase B in 6 min. The HPLC eluent was directly injected into a Thermo Scientific Quantiva triple quadrupole mass spectrometer, and amino acids were ionized using negative-ion electrospray ionization (ESI). The collected amino acid metabolites were analyzed using the Multiple Reaction Monitoring (MRM) mode with the following transitions; 4-OH-Proline: 132 > 68, Alanine: 90 > 62, Arginine: 175 > 116, Asparagine: 133 > 87, Aspartate: 134 > 88, Cysteine: 122 > 76, Cystine: 241 > 122, Glutamate: 148 > 102, Glutamine: 147 > 84, Glycine: 76 > 48, Histidine: 156 > 93, Isoleucine: 132 > 86, Leucine: 132 > 86, Lysine: 147 > 84, Methionine: 150 > 104, Phenylalanine: 166 > 120, Phenylalanine-d5: 171 > 125 (internal standard), Proline: 116 > 70, Serine: 106 > 60, Threonine: 120 > 91, Tryptophan: 205 > 146, Tyrosine: 182 > 136, and Valine: 118 > 72. The peak areas for the amino acid compounds and the internal standards were integrated using the software TraceFinder 3.1 (ThermoScientific). The internal standard calibration curves were used for the quantitation of amino acid compounds, and the levels were normalized to the protein content.

### 2.7. Statistics

All statistical analysis was performed using GraphPad Prism v8 (GraphPad Software, LLC, MacOS version 9, San Diego, CA, USA). All experiments were run in triplicate, unless otherwise indicated. The values reported in the results are the mean values ± standard error of the mean. One-way analysis of variance was used to calculate statistical significance, and the *p*-values are detailed in the text and figure legends. A *p*-value of <0.05 was considered statistically significant for all experiments.

For the metabolomics analysis, the spectral features of the MSDIAL-processed data were further analyzed via MetaboAnalyst 5.0 (www.metaboanalyst.ca, accessed on 3 October 2022). Missing values were replaced by 1/5th of the minimum positive values of their corresponding variables. A two-sample *t*-test assuming equal variances was used to calculate the *p*-values with a significance value of <0.05 and adjusted using the Benjamin–Hochberg method. The data were log transformed and fold-change analysis was performed. Multivariate principal component analysis (PCA) and hierarchical clustering were performed to understand the metabolite variation and expression patterns.

### 2.8. Animal Study Approvals

All mouse studies were completed in accordance with the Institutional Animal Care and Use Committee guidelines, approval # 2018-2003. All studies utilizing lentiviral particle generation were completed in accordance with the Institutional Biosafety Committee guidelines, approval #IBC0920.

## 3. Results

### 3.1. Ketogenic Diet in C57Bl/6mice Bearing ID8 Tumors Induced Ketosis and Increased Fat Mass

**Figure 1:**
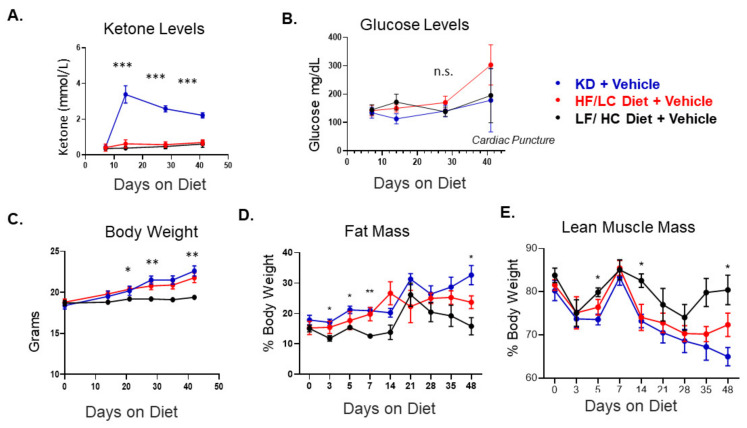
Ketogenic diet in C57Bl/6 mice bearing ID8 tumors induces ketosis and alters body composition. (**A**) Circulating ketone glucose in each diet arm with the vehicle control treatment. (**B**) Circulating glucose levels at day 42 showing elevated glucose levels due to cardiac puncture and non-significant difference in glucose levels up to day 28. (**C**) Mouse weight over the study course between the three diet groups among the vehicle-treated mice. The LF/HC-fed mice have a significantly lower weight than the KD- and HF/LC-fed mice. (**D**) Distribution of fat mass based on EchoMRI between the diet groups over the study course. (**E**) Lean muscle mass based on EchoMRI measurements between the diet groups over the study course (n = 10 mice per group, ANOVA *** *p* < 0.001, ** *p* < 0.01, and * *p* < 0.05; n.s.= not significant).

To determine the impact of KD on EOC growth, a total of 60 female C57BL/6J (BL/6) mice were injected IP with ID8 Luc cells (5 × 10^6^). One week following the injection, the mice were randomized to the LF/HC diet, HF/LC diet, and KD (20 mice per arm), followed by treatment with vehicle (control) IP injection or cisplatin chemotherapy (10 mice per arm) on a weekly schedule (Scheme 1). Within one week, circulating ketone levels were higher in the KD-fed mice compared to the HF/LC- and LF/HC-fed mice, confirming the occurrence of ketosis without significant elevation or decrease in glucose levels (Figure 1A). Interestingly, the mice fed the HF/LC diet had higher endpoint glucose levels. Between the KD- and HF/LC-fed groups, mouse body weight did not differ significantly over the course of the study (Figure 1B). However, the LF/HC-fed mice’s body weight remained stable throughout the study and was significantly lower than the KD- and HF/LC-fed mice (*p* < 0.05 and *p* < 0.01). The mice on the KD demonstrated a significant increase in fat mass and a decrease in lean muscle mass at endpoint when compared to the LF/HC- and HF/LC-treated mice based on the EchoMRI analysis, respectively (Figure 1C,D).

### 3.2. Ketogenic Diet Accelerated ID8 Ovarian Cancer Tumor Growth and Did Not Augment Response to Cisplatin Compared to Other Diet Groups

**Figure 2:**
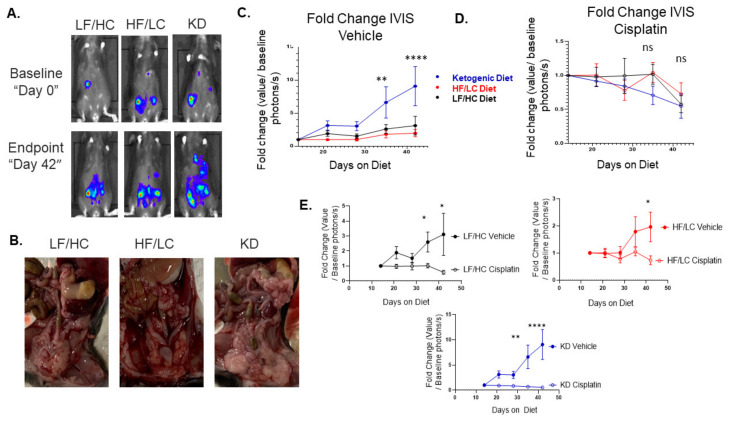
Ketogenic diet (KD) accelerates ID8 ovarian cancer tumor growth in vivo. (**A**) Biolumines-cence IVIS imaging demonstrating a significant increase in tumor growth in the ketogenic diet group over time from baseline to endpoint (Day 0). (**B**) Gross images of tumors at necropsy for each diet group. (**C**) Enhanced tumor growth on KD in vehicle treated mice compared to HF/LC diet and LF/HC diets (**** *p* < 0.0001, ** *p* < 0.01). (**D**) KD did not enhance response to chemotherapy in tumor bearing mice in vivo among the three diet groups (ns—not significant). (**E**) Fold Change IVIS vehicle vs. cisplatin: Cisplatin attenuated tumor growth compared to vehicle treated mice within each diet group. Cisplatin had most significant effect in KD group (**** *p* < 0.0001; ** *p* < 0.01; * *p* < 0.05).

The untreated, tumor-bearingKD-fed mice showed accelerated tumor growth and larger tumor burden compared to the LF/HC- and HF/LC-treated mice (Figure 2A–C). Regardless of diet, the mice treated with cisplatin exhibited decreased overall tumor burden, but without significant difference from baseline between the diet groups (Figure 2D). Interestingly, when comparing cisplatin effect within each diet group, the KD-fed mice had a significant decrease in tumor growth with the cisplatin treatment compared to the vehicle treatment, and a similar effect was seen in the LF/HC- and HF/LC-fed groups (*p* < 0.0001) (Figure 2E). Mouse weights are illustrated in supplemental Figure S1. Notably, the KD-treated mice had a higher tumor burden. Compared to the vehicle treatment, the cisplatin-treated mice maintained a similar weight until day 32 when weight appeared to decline in the cisplatin-treated mice in each of the treatment groups (HF/LC, LF/HC, and KD) (Figure S1A–C). Cumulatively, there was an overall trend toward decreased weight in the cisplatin treatment arm over the course of the study, but there was no statistically significant difference in weight among the three diet arms (Figure S1D).

### 3.3. Ketogenic Diet Was Associated with Enrichment of PPAR Signaling, Fatty Acid Metabolism, and Insulin Signaling

**Figure 3:**
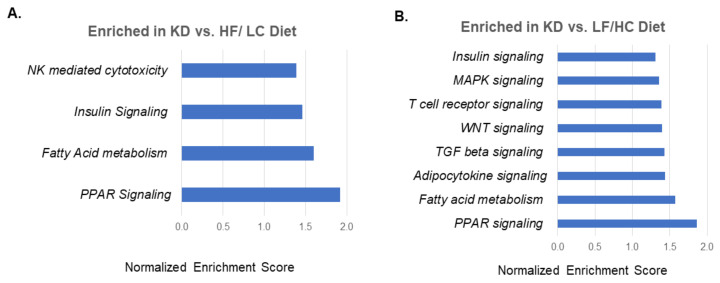
Gene Set Enrichment Analysis (GSEA) showing pathways enriched in ketogenic diet treated tumors. (**A**) GSEA normalized enrichment score for select KEGG pathways with *p*-values less than 0.05 and false discovery rate less than 0.25 in ketogenic diet vs. HF/LC diet. (**B**) Gene set enrichment analysis normalized enrichment score for select KEGG pathways with *p*-values less than 0.05 and false discovery rate less than 0.25 in KD vs. LF/HC diet.

The RNA sequencing of ID8 tumors from the mice treated with KD, HF/LC diet, and LF/HC diet were analyzed to identify the impact of diets (three tumor samples per treatment arm). The heat maps are illustrated in Figure S2A–C. GSEA-normalized enrichment score for selected KEGG pathways with *p*-values less than 0.05 and false discovery rate less than 0.25 are shown in Figure 3A,B. The pathways associated with PPAR signaling, fatty acid metabolism, insulin signaling, and natural killer (NK)-mediated cytotoxicity were enriched in the tumors of the KD-fed mice compared to the tumors of the mice fed the HF/LC diet (Figure 3A). The tumors from the KD-fed mice compared to the tumors of the mice fed the LF/HC diet showed a similar expression to the comparison of the KD vs. HF/LC diet, with additional increased levels of adipocytokine, TGF beta, WNT g, T cell receptor, and MAPK signaling. Figures S3 and S4 illustrate the heat maps of the pertinent enriched genes in the KD treatment arms.

### 3.4. KetKetogenic Diet Was Associated with Upregulation of Fatty Acid Metabolism Genes and Downregulation of Lipid Synthesis Genes

**Figure 4:**
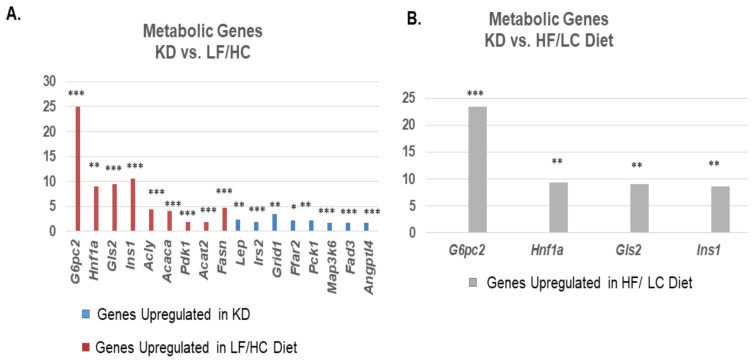
Upregulation of fatty acid metabolism genes and downregulation in lipid synthesis genes associated with ketogenic diet. (**A**) Comparison of metabolic genes between KD and LF/HC diet. Bar plots showing relevant differentially expressed metabolic genes with genes upregulated in KDshown in BLUE, and genes upregulated in LF/HC diet in ORANGE. (**B**) Comparison of differentially expressed metabolic genes between ketogenic diet and high fat/ low carbohydrate diet. Bar plots show metabolic genes upregulated in HF/LC compared to KD. (*** *p* < 0.001; ** *p* < 0.01; * *p* < 0.05).

Differentially expressed metabolic genes were compared between the ketogenic diet and LF/HC diet (Figure 4A). The genes significantly upregulated in the KD-fed mice compared to the LF/HC-fed mice included leptin (*Lep*), free fatty acid receptor 2 (*Ffar2*), and fatty acid desaturase 3 (*Fad3*), which are associated with fatty acid metabolism. Other notable metabolic genes upregulated in the KD-fed mice compared to the LF/HC-fed mice were phosphoenolpyruvate kinase 1 (*Pck1*), a key gluconeogenesis protein, insulin receptor substrate (IRS2), and glutamine receptor delta 1 subunit (GRID1). In contrast, genes associated with lipid synthesis were significantly upregulated in the LF/HC-treated tumors compared to the KD-treated tumors, including fatty acid synthetase (FASN), acetyl-CoA acetyltransferase (*Acat2*), ATP citrate lyase (*Acly*), acetyl-CoA carboxylase (*Acaca*), and pyruvate dehydrogenase kinase (PDK1) (Figure 4B). A comparison of metabolic genes between the KD- and HF/LC-treated mice showed significant upregulation of glucose 6 phosphatase catalytic subunit 2 (*G6pc2*), HNF1a, glutaminase 2 (*Gls2*), and insulin 1 (*Ins1*) in the HF/LC-treated group compared to the KD-treated group, while the remainder of genes were not significantly upregulated in either group (Figure 4B). These four genes were similarly upregulated in the LF/HC-treated group compared to KD-treated group, as shown in Figure 4A. 

### 3.5. GGlutamine/Glutamate and Fatty Acid Metabolite Concentrations Were Altered in the Plasma and Tissue of Mice on KD Compared to HF/LC Diet

**Figure 5:**
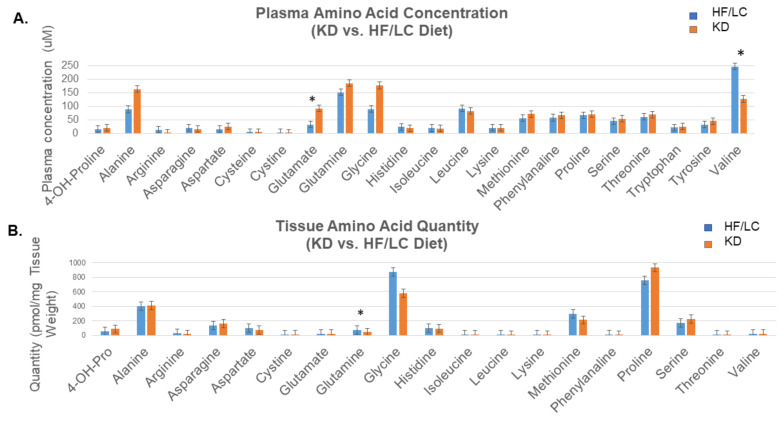
Elevated plasma glutamate and significant decrease in tissue glutamine on ketogenic diet compared to high fat/ low carbohydrate diet. (**A**) Targeted plasma amino acid metabolomics showing significant increase in plasma glutamate and a decrease in valine concentrations in KD compared to HF/LC (* *p* = 0.017 and 0.043, respectively). (**B**) Targeted tissue amino acid metabolite levels showing significant decrease in glutamine concentration KD compared to HF/LC (* *p* = 0.0007).

The targeted amino acid metabolomics analysis revealed a significant increase in plasma glutamate and a decrease in valine concentrations in the KD-treated mice compared to the HF/LC-treated mice (*p* = 0.017 and 0.043, respectively). The tissue metabolite levels of glutamine were significantly diminished in the KD-treated mice compared to the HF/LC-treated mice (*p* = 0.0007) (Figure 5). The free fatty acid concentrations in plasma are shown in Table S1. The analysis demonstrated a significant increase in free fatty acids 22:0 and 24:0 (*p* = 0.046 and 0.024) in the mice fed the KD vs. the mice fed the HF/LC diet. The tissue free fatty acid concentrations are shown in Table S3. Fatty acids 14:0 and 16:0 were significantly elevated in the tumors of the KD-treated mice compared to the tumors of the HF/LC-treated mice.

## 4. Discussion

Ketogenic diets have been shown to provide tumor inhibitory benefits for selected tumors in both animal models and human studies [33,34,35]. Herein, we show that KD promotes EOC growth when administered in an unrestricted fashion in vivo and does not augment response to cisplatin compared to HF/LC and LF/HC diets. KD induces characteristic changes in the metabolic signaling pathways that regulate fatty acid metabolism and glucose homeostasis. KD also leads to significant shifts in amino acid and fatty acid metabolite concentrations in the plasma of KD-treated mice. These results underscore the complexity of predicting the effects of acute dietary alterations and nutrient deprivation on tumor growth and suggest that tumor response to KD may be influenced by metabolic dependencies of tumors. 

We noted a global shift in metabolic pathways in the tumors of the mice treated with KD in favor of fatty acid metabolism (upregulation of *Fad3* and *Ffar2*) and a particular enrichment in adipocytokine signaling as demonstrated by upregulation of the adipocytokine leptin and the adipokine *Angptl4*. In contrast, the tumors of the mice treated with the LF/HC diet demonstrated upregulation of gluconeogenesis genes (*G4pc2*, *Acly*, *Acaca*, *Pdk1*, and *Acat*) and genes associated with fatty acid synthesis, including *Fasn*. Both *Lep* and *Angptl4* are involved in the regulation of lipid metabolism and have been implicated in ovarian cancer tumorigenesis [36,37]. Moreover, the enrichment in the PPAR signaling pathway, which regulates glucose, ketone body, and lipid metabolism, is noteworthy in the KD-treated tumors. PPARs, a family of lipid biosensors, have been shown to upregulate the expression of AGPTL4 under conditions of hypoxia and fasting [38]. ANGPTL4 promotes angiogenesis, invasion, and metastasis, and its silencing in ovarian cancer cells has been shown to delay tumor progression [38]. Leptin is similarly known to have proangiogenic and proliferative effects on ovarian cancer and promotes tumor progression [39]. The impact of altered fatty acid metabolism on ovarian cancer growth has been well described. Cancer-associated adipocytes promote ovarian cancer growth through the metabolism of fatty acids derived from and stored within adipocytes, fatty acid oxidation, and the production and secretion of cytokines [40,41]. Therefore, the induction of fatty acid metabolism genes and pathways regulating lipid metabolism is a plausible mechanism by which KD exerts its tumor-promoting effects, and this observation warrants further investigation. 

A HF/LC diet reprograms the utilization of fat in the tumor microenvironment by upregulating genes associated with lipid transport and fatty acid beta oxidation [42]. This phenomenon was observed in our studies where we noted a significant enrichment in PPAR signaling, insulin signaling, and fatty acid metabolism in the tumors of the mice treated with KD. This effect was not observed in the HF/LC-treated mice. We demonstrated a significant elevation in plasma ketone bodies in the KD-treated mice, confirming ketosis under the setting of 90% fat and 0% carbohydrate consumption, which was not observed in the mice fed with the HF/LC diet despite a difference of 15% in fat concentration. Ketone bodies have been shown in some cancers to protect from tumor growth, likely due to the inhibition of histone deacetylase as well as alterations in immune regulation [43]. More recently, it has been shown that pancreatic adenocarcinoma cells rely on ketone bodies for metabolic needs and activate ketone body metabolism to promote tumor growth and progression. This finding is dependent on the dysregulation of the metabolic enzyme, HMB-CoA lyase, involved in ketogenesis [44]. The varying genetic backgrounds of tumors and the effects of altered nutrient levels on gene expression and regulation are important factors in determining response to KD and likely impacted tumor growth seen in our ovarian cancer model.

The underlying metabolic phenotype and ketolytic enzyme expression of tumors also dictates the ability of tumor cells to utilize ketone bodies under KD conditions [23,45,46]. Tumors with a glycolytic subtype respond more favorably to KD than tumors with ketone body metabolic subtypes [47]. For example, in hepatocellular carcinoma, ketolysis is activated under glucose deprivation and ketone bodies are used as fuel for tumor growth [48]. Oxidative phosphorylation represents the main metabolic pathway in EOC [49]. However, EOC cells display metabolic heterogeneity and have the ability to alter their metabolic activity and adapt to nutritional and cellular stress [25]. We detected a significant increase in plasma glutamate levels in the KD plasma samples and a decrease in tissue glutamine compared to the HF/LC samples, which suggests an increased rate of glutamate conversion and enhanced glutamine utilization in tumor tissue. As EOC cells increase their oxygen consumption during tumor proliferation, glutamine becomes the predominant fuel [50]. Glutamine metabolism is one of the hallmarks of ovarian cancer and is known to stimulate the mTOR/MAPK and STAT3 signaling pathways, and inhibition of glutamine metabolism has been shown to provide therapeutic benefits [50,51]. Taken together, these data suggest that KD induces metabolic shifts, which increase the utilization of fatty acids, ketone bodies, and glutamine to potentially fuel tumor growth. Further exploration of this hypothesis is needed.

Our studies have some limitations. First, we were not able to control the amount of dietary intake or calorie consumption in the mice used in our studies. Thus, it is possible that ad libitum intake could have contributed to tumor growth upon a KD in accordance with prior studies [6,16]. Yet, in clinical practice, calorie restriction is not typically practiced by patients starting a KD. Additionally, we noted an increase in fat mass, an increase in ketone bodies, and no reduction in glucose levels in our studies, which was likely a result of unrestricted KD feeding. Nevertheless, the mice were closely monitored for body weight and condition, and we saw consistent changes in body composition across each diet group. Although we did not measure insulin and IGF-1 levels in the KD-treated mice to determine the effects of unrestricted KD feeding on insulin sensitivity, we suspect that the increases in fat mass and tumor growth were in part stimulated by increased insulin signaling as shown on the transcriptomics analysis. Despite the limitations, our study is one of the first to demonstrate the effect of KD in a preclinical ovarian cancer model and to characterize the molecular and metabolic responses to KD.

## 5. Conclusions

Our results challenge the notion that KD is beneficial in EOC therapy and highlight the importance of having individualized, tumor-specific recommendations for dietary interventions in patients with cancer. We found that nutrient restriction with KD had a marked effect in upregulating PPAR signaling, fatty acid metabolism, and adipocytokine signaling, thus likely contributing to tumorigenesis. As patients and healthcare providers are becoming more interested in dietary and lifestyle interventions to improve cancer outcomes, it is critical to comprehensively evaluate the full downstream effects of dietary interventions both molecularly and metabolically, and to exercise caution in making dietary recommendations. We seek to gain mechanistic insight into the effect of KD on EOC metabolism in future studies based upon our results. These data are crucial in understanding how cancers respond to various diets and how diets influence tumor metabolism so that dietary interventions can be applied in cancer therapy.

## Figures and Tables

**Table 1:** Diet composition for each study group.

Components	LF/HC (TD.150345)	HF/LC (TD.160239.PWD)	Ketogenic Diet (TD.160153.PWD)
Carbohydrate	77%	15%	0%
Fat	13%	75%	90%
Protein	10%	10%	10%
Fat source (g/kg)	Crisco (25) corn oil (25)	Crisco (314) cocoa butter (107) corn oil (60.7)	Crisco (440) Cocoa butter (150) Corn Oil (85)
Calories per gram	3.7	6.7	5.7
Vitamin mix Ain-93-VX Thiamin (81%) Vitamin K	15.0 0.01 0.002	24.0 0.016 0.0032	27.0 0.018 0.0036
Casein (g/kg)	100	160	180
Cellulose (g/g)	34.9	49.3	59.2
Mineral mix (g/kg)	13.39	20.9	24.1
Other components (g/kg)	Sucrose (100.0) Corn Starch (512.5) Maltodextrin (155.0) Calcium phosphate (9.8) Calcium carbonate (5.25)	Sucrose (30.0) Corn Starch (158.0) Maltodextrin (46.5) Calcium phosphate (15.0) Calcium carbonate (8.1)	Calcium phosphate (17.6) Calcium carbonate (9.54)

## Data Availability

The datasets used and/or analyzed during the current study are available from the corresponding author upon reasonable request.

## Supplementary Materials

The following supporting information can be downloaded at https://www.mdpi.com/article/10.3390/nu15122730/s1. Figure S1: Weekly mouse weights after cisplatin treatment vs. vehicle control. A, B, C: Mouse weekly weights in vehicle- vs. cisplatin-treated mice in the ID8 mouse model. D: Weekly mouse weights in KD, HF/LC, and LF/HC diet treatment arms with cisplatin. Figure S2: Heat maps showing the top 50 differentially expressed genes in (A) LF/HC diet vs. KD, in (B) HF/LC diet vs. LF/HC diet, and in (C) KD vs. HF/LC diet. Table S1: Comparison of plasma free fatty acids between high fat and ketogenic diet samples, showing significantly increased concentration of free fatty acids 22:0 and 24:0 in KD vs. HF/LC diet. Table S2: Comparison of tissue free fatty acids between high fat and ketogenic diet, showing significantly increased quantity of free fatty acids 14:0 and 16:0 in ketogenic-treated samples. Figure S3: Gene set enrichment analysis of KD vs. LF/HC diet. Heat maps showing GSEA of ketogenic vs. LF/HC enrichment of fatty acid metabolism, PPAR signaling, adipocytokine signaling, and TGFB signaling in ketogenic diet-treated tumors compared to low fat/high carbohydrate diet-treated tumors. High expression is denoted in red, and low expression is denoted in blue. Figure S4: Gene set enrichment analysis of ketogenic diet vs. high fat/high carbohydrate diet. Heat maps showing GSEA of KD vs. HF/LC diet with enrichment of fatty acid metabolism, PPAR signaling, insulin signaling, and NK-mediated cytotoxicity in KD-compared to HF/LC diet-treated rumors. High expression is denoted in red, and low expression id denoted in blue.

## Abbreviations

*Acaca*	Acetyl-CoA carboxylase
*Acat2*	Acetyl-CoA acetyltransferase 2
*Acly*	ATP citrate lyase
Agptl4	Angiopoietin type 4
AMP	Adenosine monophosphate
ATP	Adenosine triphosphate
BCS	Body composition scoring
*Bdh1*	3-hydroxybutyrate dehydrogenase 1
*Bdh2*	3-hydroxybutyrate dehydrogenase 2
BHB	Beta hydroxybutyrate
*Cpt1*	Carnitine palmitoyltransferase
DMEM	Dulbecco’s Modified Eagle Medium
EOC	Epithelial ovarian cancer
ESI	Electrospray ionization
FA	Fatty acid
*Fad3*	Fatty acid desaturase
*Fasn*	Fatty acid synthetase
FBS	Fetal bovine serum
*Ffar2*	Free fatty acid receptor 2
*G6pc2*	Glucose 6 phosphatase catalytic subunit 2
*Gls2*	Glutaminase 2
*Grid1*	Glutamine receptor delta 1 subunit
GSEA	Gene set enrichment analysis
HF/LC	High fat/low carbohydrate
*Hnf1a*	Hepatocyte nuclear factor 1 alpha
HPLC	High-throughput liquid chromatography
IACUC	Institutional Animal Care and Use Committee
ID8-luc	ID8 luciferase cells
*Igf*	Insulin growth factor
*Ins1*	Insulin 1
IP	Intraperitoneal
*Irs2*	Insulin receptor substrate 2
IVIS	Spectrum in vivo imaging system
KD	Ketogenic diet
KEGG	Kyoto Encyclopedia of Gene and Genomes
LC-MS	Liquid chromatography–mass spectrometry
*Lep*	Leptin
LF/HC	Low fat/high carbohydrate
*Map*	Mitogen-activated protein
MRM	Multiple Reaction Monitoring
NK	Natural killer
*Oxct1*	3-Oxoacid CoA-transferase 1
*Oxct2*	3-Oxoacid CoA-transferase 1
PCA	Principal component analysis
PCK1	phosphoenolpyruvate kinase 1
PDK1	Pyruvate dehydrogenase kinase
PPAR	Peroxisome proliferator-activated receptor
RNA	Ribonucleic acid
SCD	Stearoyl-CoA desaturase
STAR	Spliced Transcripts Alignment to a Reference
STAT3	Signal transducer and activator of transcription 3
TGF	Transforming growth factor
WNT	Wingless type
