# Supplementary material for: Unrestricted Ketogenic Diet Feeding Enhances Epithelial Ovarian Cancer Growth In Vivo
Source: Nutrients. 2023 Jun 13;15(12):2730. doi: 10.3390/nu15122730 (PMC10301007; doi:10.3390/nu15122730)
Supplement: Supplementary file 1 [file nutrients-15-02730-s001.zip › nutrients-2400899-supplementary.pptx]

## Slide 1
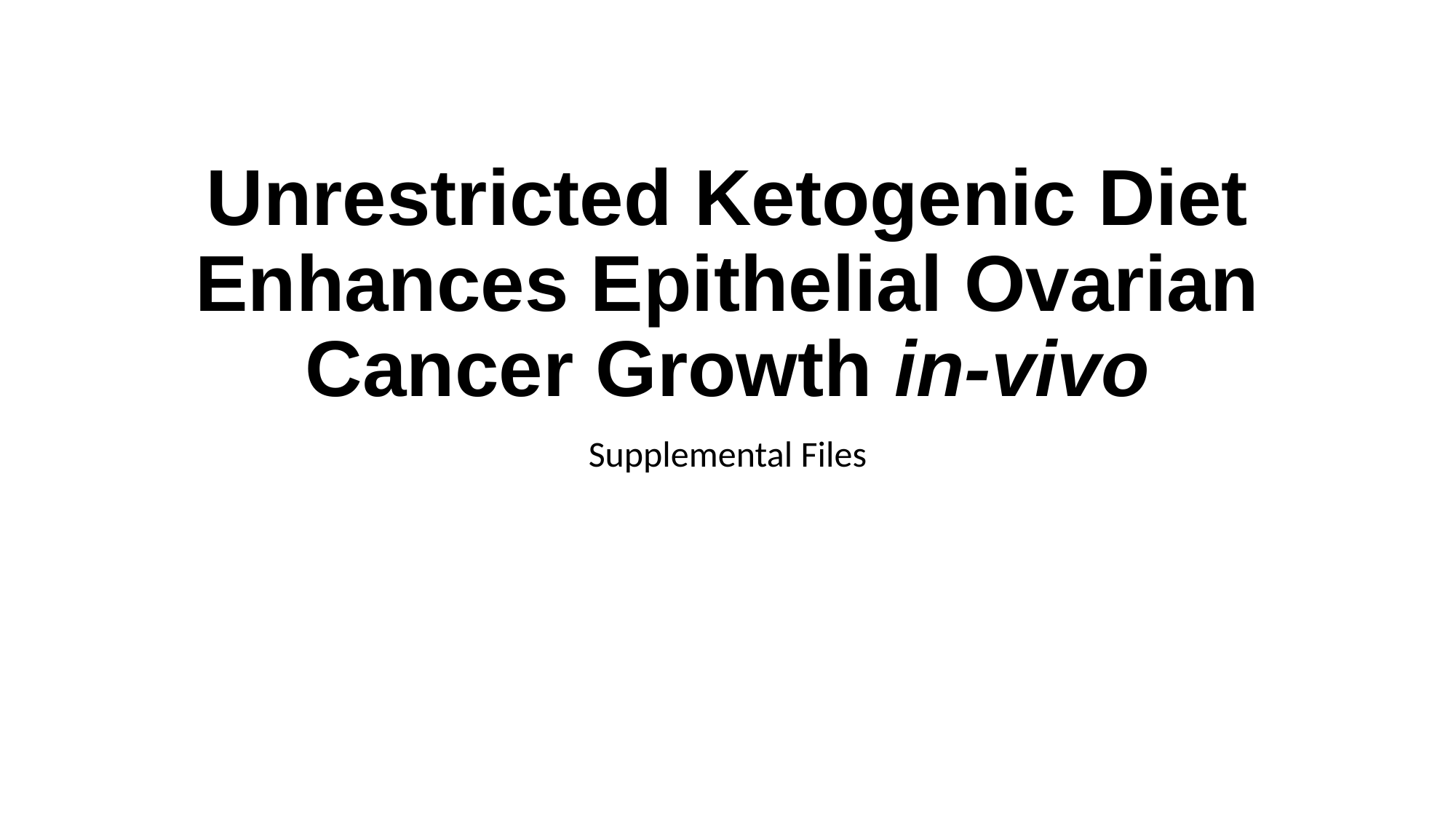

# Unrestricted Ketogenic Diet Enhances Epithelial Ovarian Cancer Growth in-vivo
Supplemental Files

## Slide 2
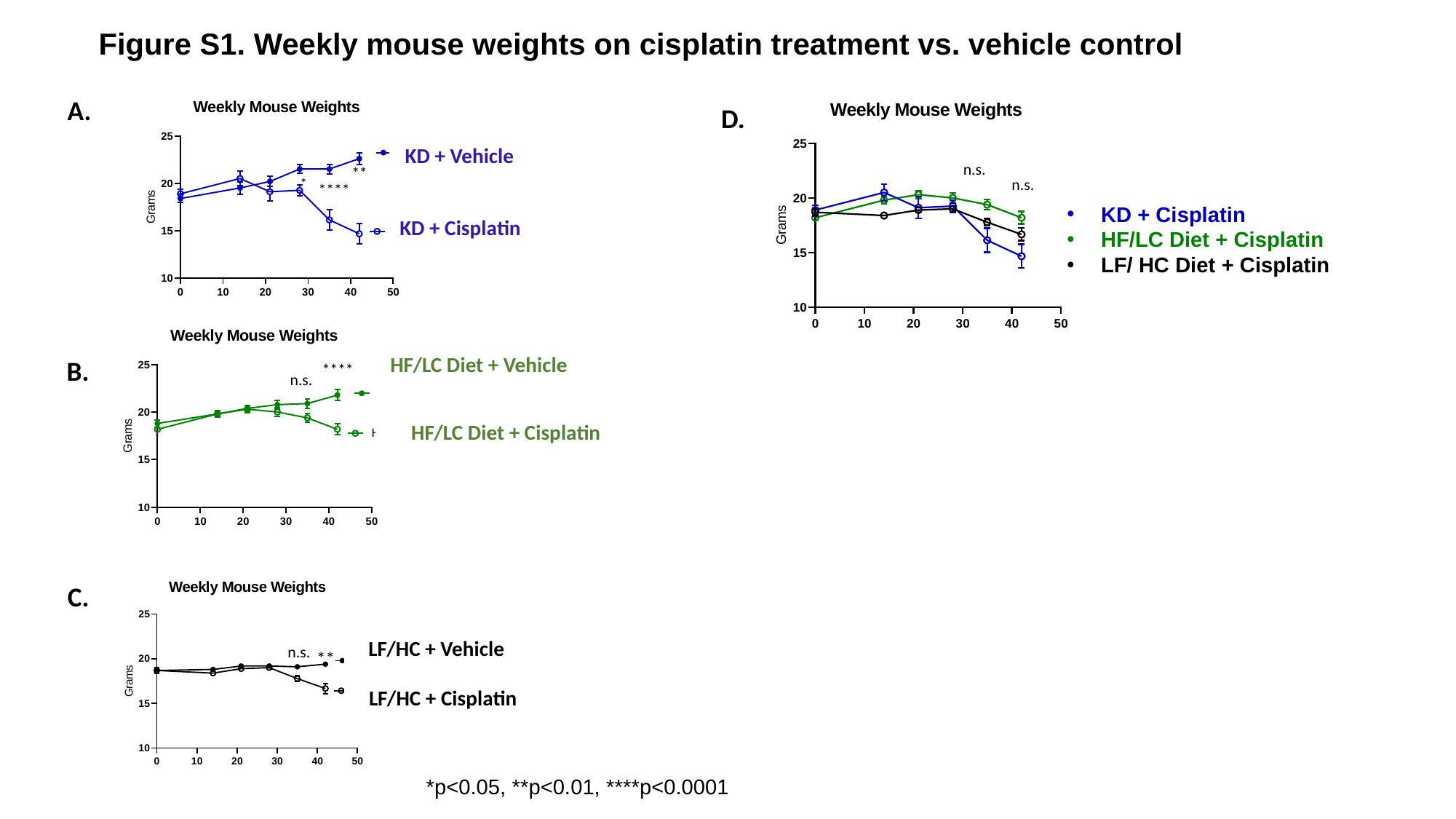

Figure S1. Weekly mouse weights on cisplatin treatment vs. vehicle control
A.
**
*
****
D.
KD + Vehicle
n.s.
n.s.
KD + Cisplatin
HF/LC Diet + Cisplatin
LF/ HC Diet + Cisplatin
KD + Cisplatin
****
n.s.
HF/LC Diet + Vehicle
B.
HF/LC Diet + Cisplatin
**
n.s.
C.
LF/HC + Vehicle
LF/HC + Cisplatin
*p<0.05, **p<0.01, ****p<0.0001

## Slide 3
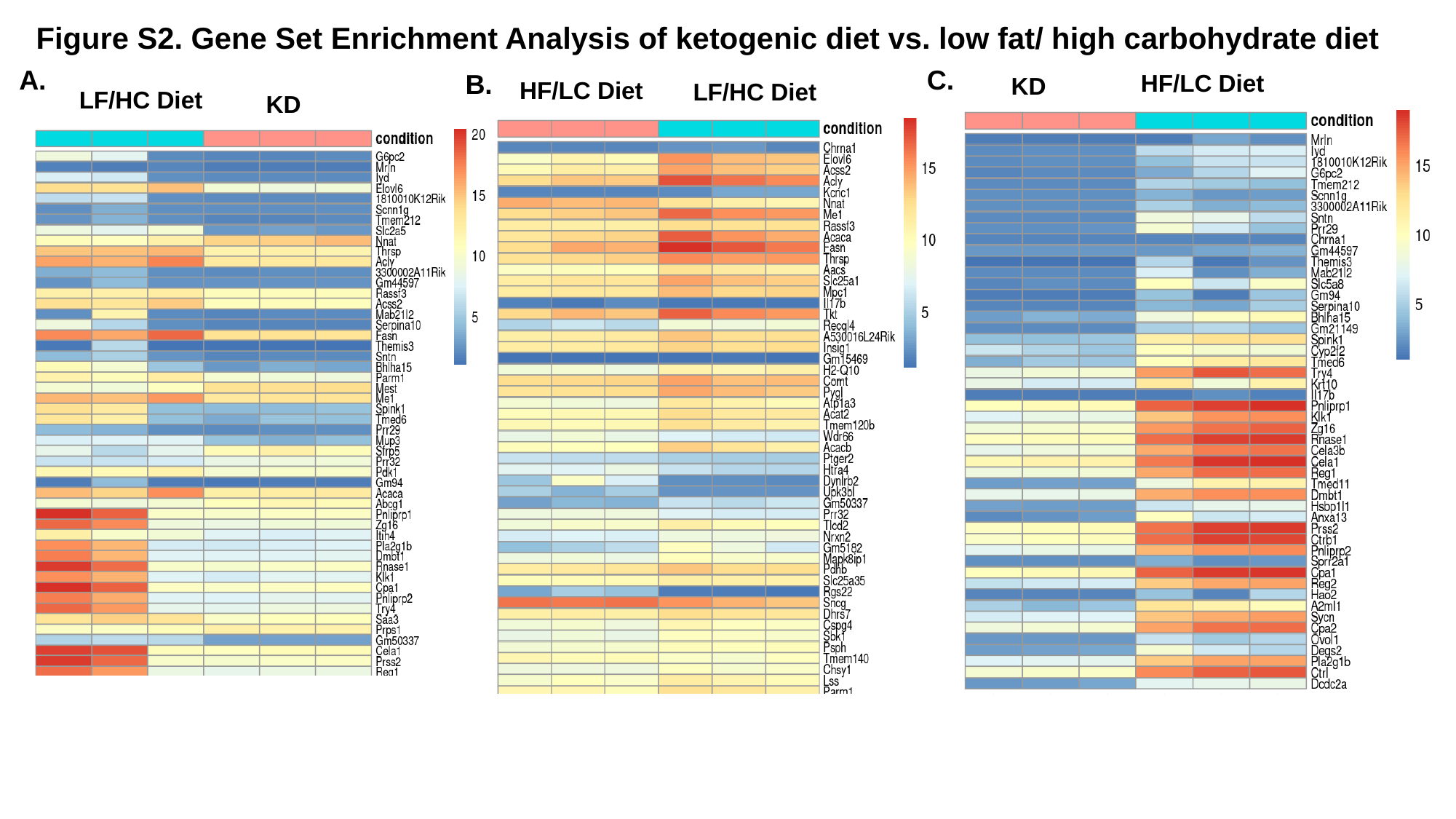

Figure S2. Gene Set Enrichment Analysis of ketogenic diet vs. low fat/ high carbohydrate diet
A.
C.
B.
HF/LC Diet
KD
HF/LC Diet
LF/HC Diet
LF/HC Diet
KD

## Slide 4
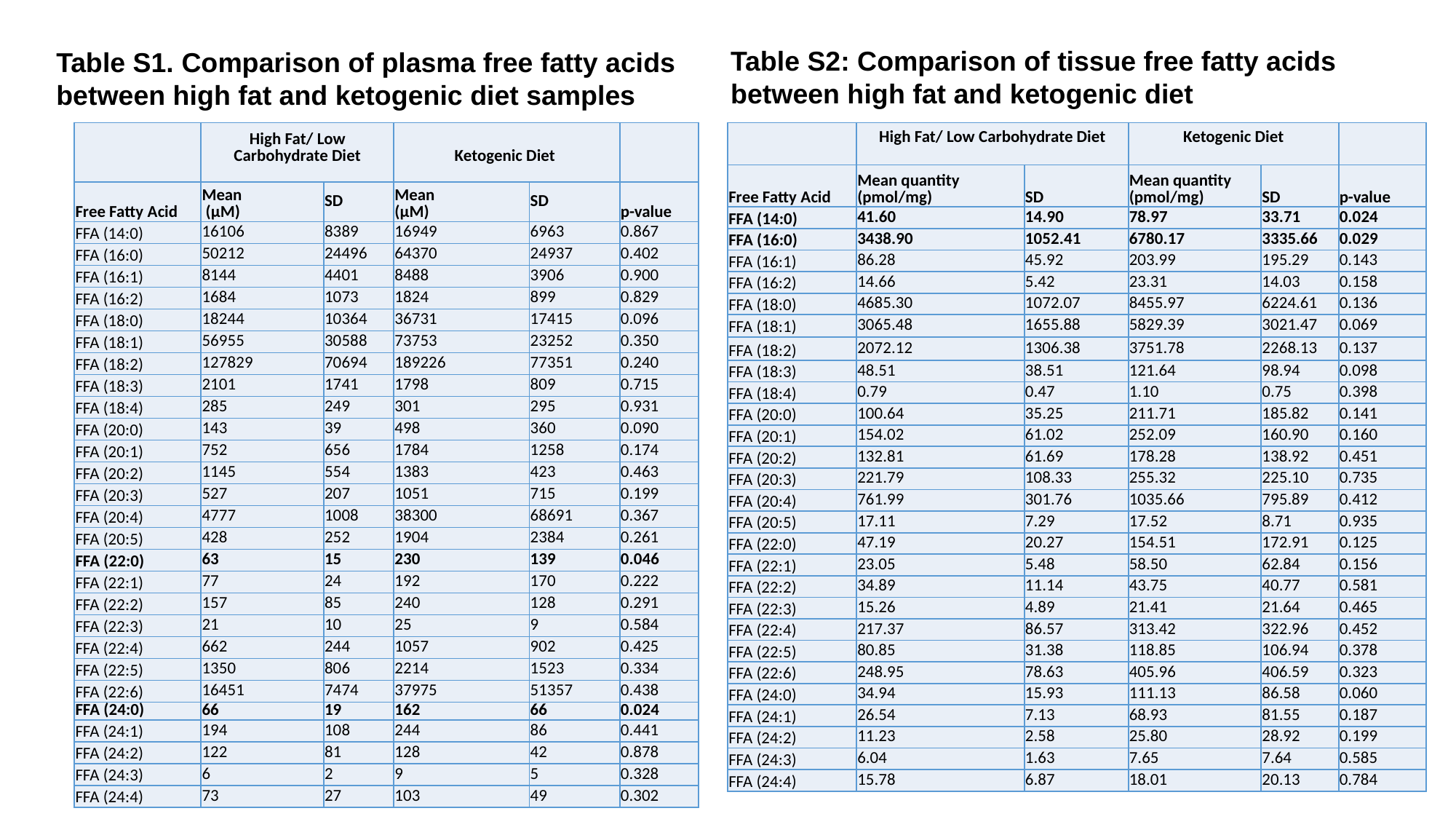

Table S2: Comparison of tissue free fatty acids between high fat and ketogenic diet
Table S1. Comparison of plasma free fatty acids between high fat and ketogenic diet samples
| | High Fat/ Low Carbohydrate Diet | | Ketogenic Diet | | |
| --- | --- | --- | --- | --- | --- |
| Free Fatty Acid | Mean (µM) | SD | Mean (µM) | SD | p-value |
| FFA (14:0) | 16106 | 8389 | 16949 | 6963 | 0.867 |
| FFA (16:0) | 50212 | 24496 | 64370 | 24937 | 0.402 |
| FFA (16:1) | 8144 | 4401 | 8488 | 3906 | 0.900 |
| FFA (16:2) | 1684 | 1073 | 1824 | 899 | 0.829 |
| FFA (18:0) | 18244 | 10364 | 36731 | 17415 | 0.096 |
| FFA (18:1) | 56955 | 30588 | 73753 | 23252 | 0.350 |
| FFA (18:2) | 127829 | 70694 | 189226 | 77351 | 0.240 |
| FFA (18:3) | 2101 | 1741 | 1798 | 809 | 0.715 |
| FFA (18:4) | 285 | 249 | 301 | 295 | 0.931 |
| FFA (20:0) | 143 | 39 | 498 | 360 | 0.090 |
| FFA (20:1) | 752 | 656 | 1784 | 1258 | 0.174 |
| FFA (20:2) | 1145 | 554 | 1383 | 423 | 0.463 |
| FFA (20:3) | 527 | 207 | 1051 | 715 | 0.199 |
| FFA (20:4) | 4777 | 1008 | 38300 | 68691 | 0.367 |
| FFA (20:5) | 428 | 252 | 1904 | 2384 | 0.261 |
| FFA (22:0) | 63 | 15 | 230 | 139 | 0.046 |
| FFA (22:1) | 77 | 24 | 192 | 170 | 0.222 |
| FFA (22:2) | 157 | 85 | 240 | 128 | 0.291 |
| FFA (22:3) | 21 | 10 | 25 | 9 | 0.584 |
| FFA (22:4) | 662 | 244 | 1057 | 902 | 0.425 |
| FFA (22:5) | 1350 | 806 | 2214 | 1523 | 0.334 |
| FFA (22:6) | 16451 | 7474 | 37975 | 51357 | 0.438 |
| FFA (24:0) | 66 | 19 | 162 | 66 | 0.024 |
| FFA (24:1) | 194 | 108 | 244 | 86 | 0.441 |
| FFA (24:2) | 122 | 81 | 128 | 42 | 0.878 |
| FFA (24:3) | 6 | 2 | 9 | 5 | 0.328 |
| FFA (24:4) | 73 | 27 | 103 | 49 | 0.302 |
| | High Fat/ Low Carbohydrate Diet | | Ketogenic Diet | | |
| --- | --- | --- | --- | --- | --- |
| Free Fatty Acid | Mean quantity (pmol/mg) | SD | Mean quantity (pmol/mg) | SD | p-value |
| FFA (14:0) | 41.60 | 14.90 | 78.97 | 33.71 | 0.024 |
| FFA (16:0) | 3438.90 | 1052.41 | 6780.17 | 3335.66 | 0.029 |
| FFA (16:1) | 86.28 | 45.92 | 203.99 | 195.29 | 0.143 |
| FFA (16:2) | 14.66 | 5.42 | 23.31 | 14.03 | 0.158 |
| FFA (18:0) | 4685.30 | 1072.07 | 8455.97 | 6224.61 | 0.136 |
| FFA (18:1) | 3065.48 | 1655.88 | 5829.39 | 3021.47 | 0.069 |
| FFA (18:2) | 2072.12 | 1306.38 | 3751.78 | 2268.13 | 0.137 |
| FFA (18:3) | 48.51 | 38.51 | 121.64 | 98.94 | 0.098 |
| FFA (18:4) | 0.79 | 0.47 | 1.10 | 0.75 | 0.398 |
| FFA (20:0) | 100.64 | 35.25 | 211.71 | 185.82 | 0.141 |
| FFA (20:1) | 154.02 | 61.02 | 252.09 | 160.90 | 0.160 |
| FFA (20:2) | 132.81 | 61.69 | 178.28 | 138.92 | 0.451 |
| FFA (20:3) | 221.79 | 108.33 | 255.32 | 225.10 | 0.735 |
| FFA (20:4) | 761.99 | 301.76 | 1035.66 | 795.89 | 0.412 |
| FFA (20:5) | 17.11 | 7.29 | 17.52 | 8.71 | 0.935 |
| FFA (22:0) | 47.19 | 20.27 | 154.51 | 172.91 | 0.125 |
| FFA (22:1) | 23.05 | 5.48 | 58.50 | 62.84 | 0.156 |
| FFA (22:2) | 34.89 | 11.14 | 43.75 | 40.77 | 0.581 |
| FFA (22:3) | 15.26 | 4.89 | 21.41 | 21.64 | 0.465 |
| FFA (22:4) | 217.37 | 86.57 | 313.42 | 322.96 | 0.452 |
| FFA (22:5) | 80.85 | 31.38 | 118.85 | 106.94 | 0.378 |
| FFA (22:6) | 248.95 | 78.63 | 405.96 | 406.59 | 0.323 |
| FFA (24:0) | 34.94 | 15.93 | 111.13 | 86.58 | 0.060 |
| FFA (24:1) | 26.54 | 7.13 | 68.93 | 81.55 | 0.187 |
| FFA (24:2) | 11.23 | 2.58 | 25.80 | 28.92 | 0.199 |
| FFA (24:3) | 6.04 | 1.63 | 7.65 | 7.64 | 0.585 |
| FFA (24:4) | 15.78 | 6.87 | 18.01 | 20.13 | 0.784 |

## Slide 5
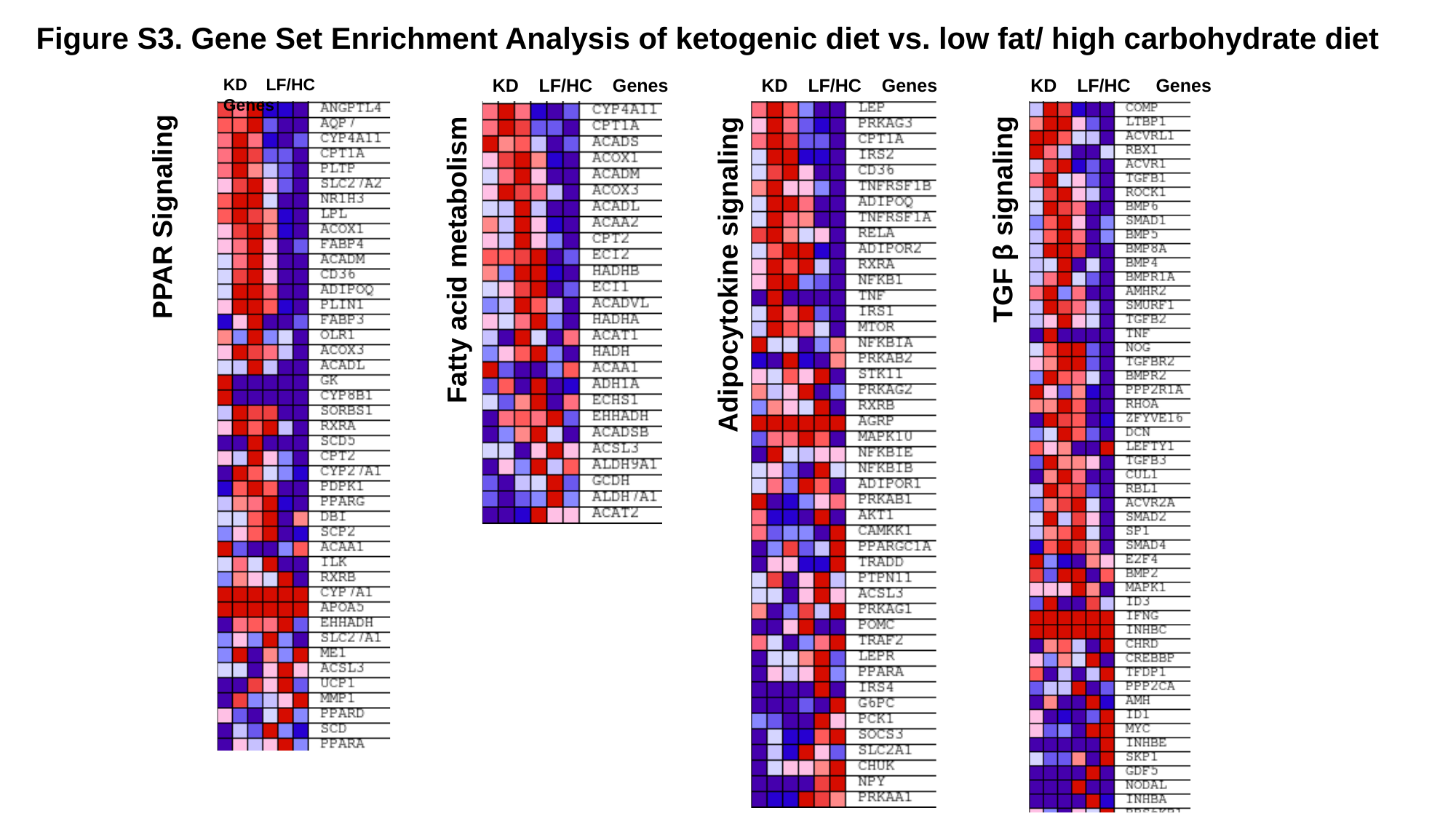

Figure S3. Gene Set Enrichment Analysis of ketogenic diet vs. low fat/ high carbohydrate diet
KD LF/HC Genes
KD LF/HC Genes
KD LF/HC Genes
KD LF/HC Genes
PPAR Signaling
TGF β signaling
Fatty acid metabolism
Adipocytokine signaling

## Slide 6
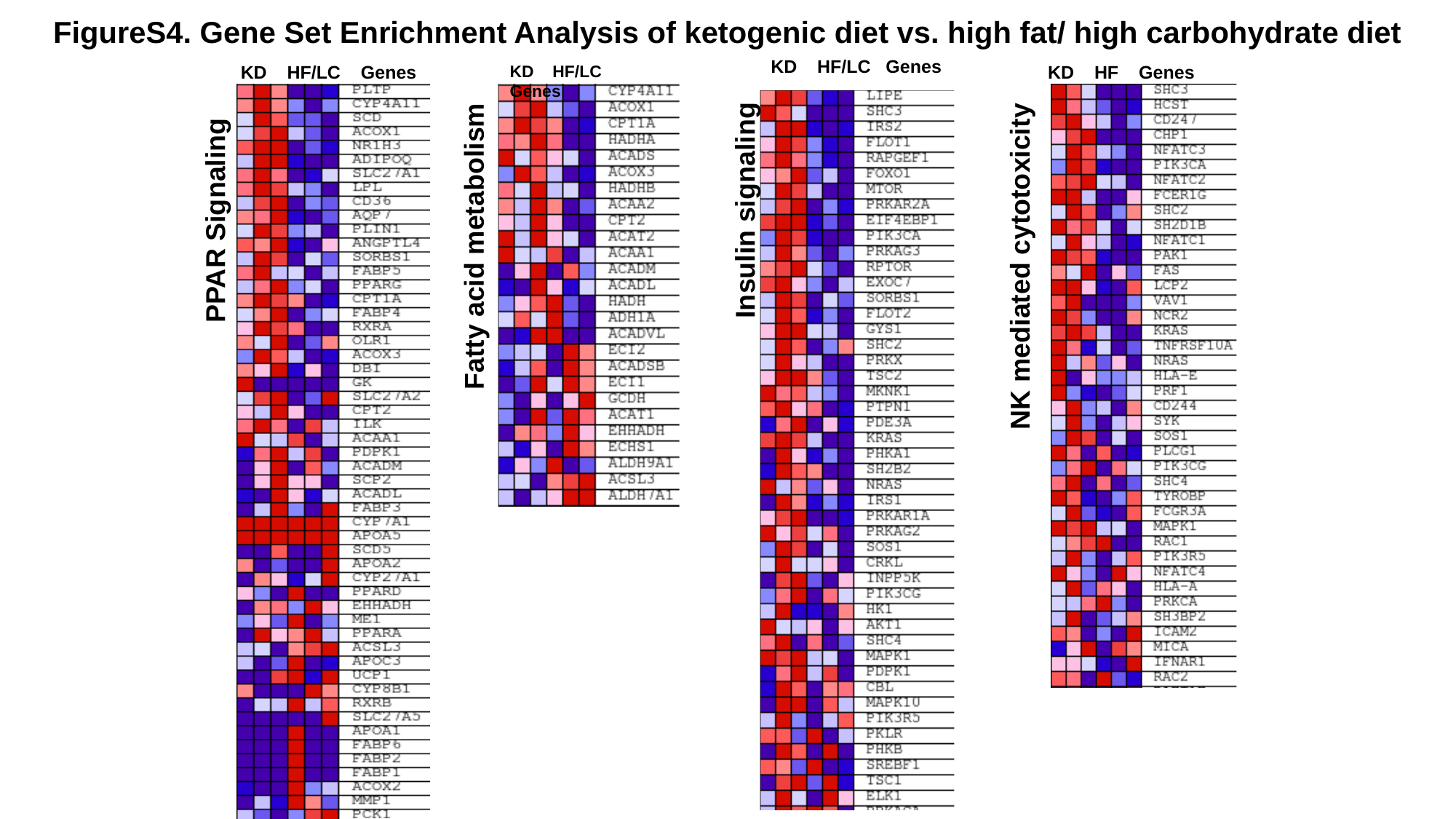

FigureS4. Gene Set Enrichment Analysis of ketogenic diet vs. high fat/ high carbohydrate diet
KD HF/LC Genes
KD HF/LC Genes
KD HF/LC Genes
KD HF Genes
Insulin signaling
PPAR Signaling
Fatty acid metabolism
NK mediated cytotoxicity
